# Experimental models and tools to tackle glioblastoma

**DOI:** 10.1242/dmm.040386

**Published:** 2019-09-01

**Authors:** Faye L. Robertson, Maria-Angeles Marqués-Torrejón, Gillian M. Morrison, Steven M. Pollard

**Affiliations:** MRC Centre for Regenerative Medicine and Edinburgh Cancer Research UK Cancer Centre, University of Edinburgh, 5 Little France Drive, Edinburgh EH16 4UU, UK

**Keywords:** Central nervous system, *In vitro*, CRISPR/Cas9, Mouse, Human, Xenograft, GBM, Cancer, Brain tumour

## Abstract

Glioblastoma multiforme (GBM) is one of the deadliest human cancers. Despite increasing knowledge of the genetic and epigenetic changes that underlie tumour initiation and growth, the prognosis for GBM patients remains dismal. Genome analysis has failed to lead to success in the clinic. Fresh approaches are needed that can stimulate new discoveries across all levels: cell-intrinsic mechanisms (transcriptional/epigenetic and metabolic), cell-cell signalling, niche and microenvironment, systemic signals, immune regulation, and tissue-level physical forces. GBMs are inherently extremely challenging: tumour detection occurs too late, and cells infiltrate widely, hiding in quiescent states behind the blood-brain barrier. The complexity of the brain tissue also provides varied and complex microenvironments that direct cancer cell fates. Phenotypic heterogeneity is therefore superimposed onto pervasive genetic heterogeneity. Despite this bleak outlook, there are reasons for optimism. A myriad of complementary, and increasingly sophisticated, experimental approaches can now be used across the research pipeline, from simple reductionist models devised to delineate molecular and cellular mechanisms, to complex animal models required for preclinical testing of new therapeutic approaches. No single model can cover the breadth of unresolved questions. This Review therefore aims to guide investigators in choosing the right model for their question. We also discuss the recent convergence of two key technologies: human stem cell and cancer stem cell culture, as well as CRISPR/Cas tools for precise genome manipulations. New functional genetic approaches in tailored models will likely fuel new discoveries, new target identification and new therapeutic strategies to tackle GBM.

## Abstract (precis)

**Summary:** This Review discusses preclinical modelling of glioblastoma multiforme to understand its biology and develop therapies, with a focus on mammalian model systems.

*“All models are wrong, but some are useful.”* – George E. P. Box.

## The challenges of glioblastoma multiforme

Glioblastoma multiforme (GBM) is the most common malignant primary brain tumour. Most cases arise sporadically. There are no effective therapies, and multi-modality treatment with surgery, radiotherapy and chemotherapy provides only ∼1 year median survival (Stupp et al., 2005). Because GBMs often arise in young adults and have poor prognosis, they account for more years of active life lost than any other cancer (Burnet et al., 2005). Together with medulloblastoma – the most common paediatric brain tumour – GBMs therefore now account for more deaths in the under 40s than any other cancer.

Gliomas are categorised as astrocytomas or oligodendrogliomas based on the predominant cell type observed on histological analysis. GBM, the most aggressive form of astrocytoma, is also, unfortunately, the most common. Its defining features are abundant mitotic cells, extensive necrosis, nuclear pleomorphism, and hyperproliferation of endothelial cells (Louis et al., 2016). A subset of patients harbour gain-of-function heterozygous mutations in isocitrate dehydrogenase (*IDH1*/*IDH2*) (Parsons et al., 2008). These IDH-mutant GBMs are the 5-10% of cases previously termed secondary GBM (Louis et al., 2016).

In children, GBMs arising in the cerebral hemispheres are often termed paediatric high-grade glioma (pHGG). When arising within the midline/brainstem, they are termed diffuse intrinsic pontine glioma (DIPG). Paediatric GBMs harbour different genetic drivers than adult tumours (e.g. *H3F3FA*, encoding histone H3.3, is mutated in pHGG and DIPG) (Mackay et al., 2017; Schwartzentruber et al., 2012; Capper et al., 2018). Fortunately, these paediatric and young-adult tumours are rare.

**Fig. 1.**
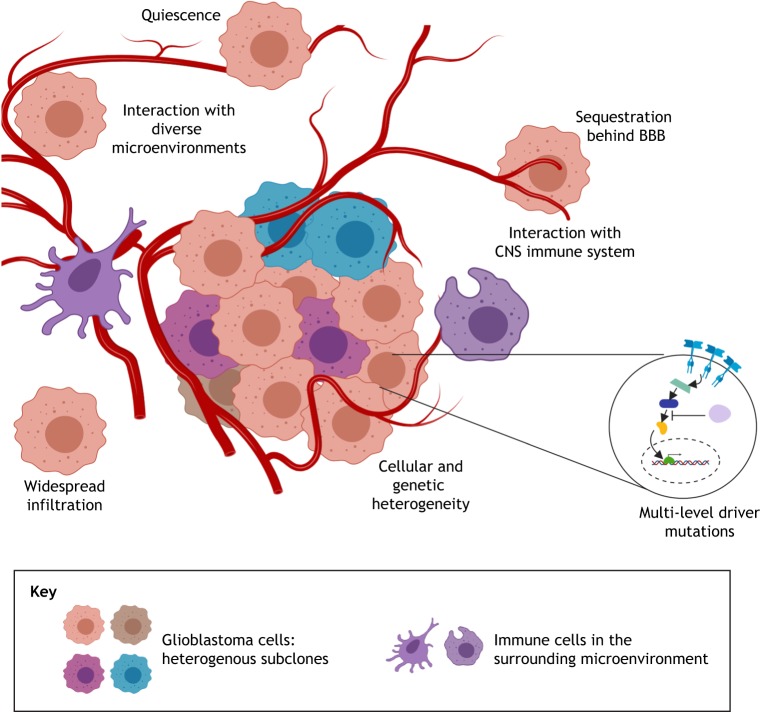
**Important challenges in understanding the biology of GBM.** GBM stem cells exist in various states (dormant/quiescent, activated/quiescent or proliferative) that are influenced by diverse tumour microenvironments (TMEs). Complex niches, immune interactions and physical forces/mechanosignalling are all poorly understood areas of GBM biology. How these influence tumour cell signalling circuits and the subsequent transcriptional and epigenetic changes in GSC fate is an area of active research. Targeting both the quiescent and proliferative tumour populations will be vital for any successful therapeutic strategy.

Why has it been so challenging to develop effective treatments for GBM? There are many inherent challenges (Fig. 1): (1) GBMs are often detected late and display extensive cellular and genetic heterogeneity; (2) driver mutations occur at many levels within canonical cell-growth and -survival pathways, undermining the approach of ‘drugging’ a single oncogenic protein; (3) tumour cells disperse widely in the brain parenchyma, limiting possibilities for surgical resection; (4) tumour cells interact with diverse and complex brain microenvironments (Quail and Joyce, 2017), often existing in dormant or quiescent states that are resistant to cytotoxic therapies (Chen et al., 2012a); (5) the blood-brain barrier (BBB) limits drug bioavailability and facilitates immune evasion; and (6) branched Darwinian evolution within the tumour creates diverse subclonal variants that undermine targeted therapies and drive relapse. Many other factors, including those related to how we operate as a research community, have also hindered progress, with barriers to progress across the whole research and clinical pipeline (Aldape et al., 2019).

Here, we discuss the range of experimental models and tools that can be deployed both to study the biology of GBM and to underpin the search for new therapeutics. We summarise the contributions that current models have made to our understanding of these tumours and the avenues being explored to develop new therapies, focussing on mammalian models. Non-mammalian models also clearly have value in helping to dissect the key mechanisms and are summarised in Box 1. We do not attempt to provide an exhaustive review of GBM biology and preclinical studies; rather, we aim to present exemplars of the available models and strategies, which increasingly can be combined and readily deployed by individual labs.

**Box 1. Non-mammalian models of glioblastoma**

Non-mammalian models also provide great value in exploring glioblastoma, although space constraints have limited our discussion here. The fly and worm – *Drosophila melanogaster* and *Caenorhabditis* *elegans* – provide a cost-effective alternative to mammalian studies that are easy to handle and have an armoury of established and high-level genetic tools. These have particular value for applications in unbiased genetic screens and related clonal lineage analysis. Many molecular pathways are conserved between *Drosophila* and human, and models of glioma have been generated in *Drosophila* in which EGFR-Ras and PI3K pathways drive neoplastic glial growths that are transplantable (Read et al., 2009). *Drosophila* researchers have a long history of making key discoveries in developmental neurobiology, particularly the mechanism of cell fate and differentiation by neural stem and progenitor cells (Jacob et al., 2008; Sousa-Nunes et al., 2010).

Zebrafish also provide unique opportunities for exploring GBM (Pudelko et al., 2018). The transparency of the fish allows elegant imaging studies, visualising tumour cell behaviours and host tissue interactions, e.g. microglia–tumour-cell interactions (Hamilton et al., 2016). Zebrafish is also an incredibly valuable vertebrate model for performing forward genetic screens, and recent CRISPR tools (Prykhozhij and Berman, 2018) are opening up possibilities for reverse genetic approaches. In coming years, the ability to perform chemical and genetic screens in zebrafish embryos and young adults in medium throughput should complement the drug discovery efforts. It is noteworthy that zebrafish is well suited for applications along the drug discovery and development pipeline, particularly during the hit-to-lead stages where assessing compound delivery, toxicities and target specificity can all be rapidly and cheaply explored in a whole vertebrate organism at scale (Stewart et al., 2014).

We also look ahead to the many new and emerging tools. The advent of CRISPR-based genome engineering, stem-cell-culture paradigms and high-content phenotypic screening are stimulating new approaches to functional genetic dissection and drug discovery efforts (O'Duibhir and Pollard, 2017). Few other human cancers have such a wealth of tractable experimental models as GBM does. These will now need to be exploited to drive new discoveries and innovations in therapeutic strategies.

## The need for tractable experimental models

The question of why we need models is perhaps self-evident: to explore the fundamental biology and test therapeutics in a way that is not possible by working directly with human patients. It is perhaps useful to draw a distinction between two types of experimental model: those designed from a reductionist viewpoint, or alternatively those that embrace and try to recapitulate the ‘real’ disease complexity. Reductionist models provide a shortcut to decisive mechanistic insights by focussing on specific aspects of tumour biology (e.g. cells in culture as material for biochemical studies), but thereby risk having limited disease relevance. An ideal reductionist experimental model benefits from being as simple as possible to ensure reliable mechanistic and functional insights; these might often focus on one particular feature (e.g. *in vitro* studies can provide new insights into cell cycle control, even though host-tumour interactions or infiltration cannot be explored). By contrast, when the goal is testing of therapeutic strategies, it often becomes critical that models closely mimic the human disease situation, with all the associated complexity. The more complex the model, the less straightforward it will be to dissect clear mechanisms because of increased heterogeneity and diversity of signals, and a larger range of tumour cell states. Investigators therefore need to balance the inevitable trade-offs in selecting a model that best fits their research question.

## Knowing your enemy: the molecular and cellular aetiology of GBM

In order to model GBM effectively, we must understand both the mutations and the epigenetic disruptions that lead to tumorigenesis and engineer these into a disease-relevant cell of origin. GBM has been extensively characterised using large-scale sequencing of its exome, genome, transcriptome and epigenome (Brennan et al., 2013; Cancer Genome Atlas Research Network, 2008; Capper et al., 2018; Sturm et al., 2012; Verhaak et al., 2010). These and related studies have revealed the simultaneous disruption of core cell cycle, growth and survival pathways as major drivers of adult GBM. Frequent gain-of-function mutations resulting from amplifications, insertions/deletions or somatic activating point mutations are seen for *EGFR*, *MET* and *PDGFRA*. These alterations stimulate the downstream RAS/ERK and phosphoinositide-3-kinase (PI3K)/AKT signalling pathways. Loss of the tumour suppressors *CDKN2A*, *TP53*, *RB*, *PTEN* and *NF1* is also frequently observed. More recent work identified mutations in the *TERT* promoter across the majority of GBMs (76% of IDH wild-type GBM cases) (Eckel-Passow et al., 2015).

Epigenetic regulators – chromatin modifiers, remodellers, histone variants and the DNA methylation apparatus – are also a category of frequently disrupted genes in adult and paediatric GBM (Brennan et al., 2013). These were initially overlooked due to low-frequency mutations across many individual genes that nevertheless disrupt the same multiprotein complexes (e.g. BAF/PBAF) (Brennan et al., 2013). Disruption of the core transcriptional and epigenetic machinery therefore seems to be a general feature of GBMs (Mack et al., 2015). GBMs also invariably display chromosome instability, with whole-chromosome gains and losses, and are therefore highly aneuploid with diverse and dynamic karyotypes.

GBM has a high degree of genetic heterogeneity, both within and between tumours. Distinct oncogenes are amplified in a mosaic and often mutually exclusive manner within a single tumour, co-existing within intermingled subclonal populations (Snuderl et al., 2011). This formidable level of heterogeneity has inevitably hampered targeted therapies against these pathways. Also, EGFR, as well as other oncogenic drivers (PDGFR and MET), are often activated in different ways within the same tumour (Furnari et al., 2015). Branched evolutionary processes further contribute to the heterogeneity (Piccirillo et al., 2015), and so interventions against key molecular targets may well need to be focused on truncal mutations. Oncogene amplification often takes the form of extrachromosomal DNA, which underlies rapid shifts in copy number (Turner et al., 2017). Tumour cells are therefore neither monoclonal nor monogenetic and exploit strategies that enable rapid adaptation due to constant genomic diversity – this is more akin to prokaryotic-like mechanisms (Verhaak et al., 2019).

Researchers have also used transcriptional profiling to catalogue the diversity of GBMs in an attempt to understand tumour heterogeneity. This work led to the proposal of three tumour-cell-intrinsic transcriptional signatures – classical, proneural and mesenchymal – with a fourth previously reported ‘neural’ subtype dismissed (Wang et al., 2017). However, single-cell analysis of GBM specimens has shown that these subtypes are not mutually exclusive, with cells from the same patients' tumours expressing distinct expression signatures (Patel et al., 2014). Therefore, instead of thinking of these subtypes as discrete disease entities, it is perhaps more helpful to view them as shifting developmental states, with differentiation biases influenced by extrinsic or intrinsic cues. Thus, while very valuable for exploring the biology of the disease, transcriptional signatures are currently less valuable as clinical or prognostic markers.

A major shift in our views of the aetiology of GBM resulted from an improved understanding of the biology of neural development, particularly the identity of neural stem cells (NSCs) and progenitor cells. Many of the key markers that emerged in the 1990s, such as nestin (*Nes*) (Lendahl et al., 1990), were found to be widely expressed in gliomas (Dahlstrand et al., 1992). CD133 (Uchida et al., 2000), a cell-surface epitope enriched in NSCs, was also used in critical functional studies that isolated a subset of GBM cells with enhanced tumour-initiation capacity compared to the CD133-negative population (Singh et al., 2004). These findings support the cancer-stem-cell model for GBM, with subsets of tumour cells displaying NSC markers and these being more aggressive than their more differentiated progeny. Recent studies, using *in vivo* genetic-lineage tracing in xenografts, lend further support to a differentiation hierarchy of GBM cells, and a subset of cells have higher clonogenic output (Lan et al., 2017).

These discoveries raise the related question of whether NSCs are a likely cell of origin (Chen et al., 2012b). Human subventricular zone (SVZ) astrocytes with germinal activity have been reported in the adult forebrain ventricles (Sanai et al., 2004); however, whether NSCs persist into adulthood within the human hippocampus remains controversial (Moreno-Jiménez et al., 2019; Sorrells et al., 2018). Analysis of primary human GBM specimens suggests that truncal driver mutations are indeed present within the adult NSC niche – the SVZ – in many patients, in tissue that is macroscopically normal (Lee et al., 2018). Several mouse studies have also indicated that SVZ stem cells are more easily transformed than astrocytes (discussed in the sections below). Unfortunately, much confusion has arisen due to the fact that differentiated astrocytes and endogenous adult NSCs (‘type B’ cells) share many markers, including GFAP (Doetsch et al., 1999). Additionally, oligodendrocyte progenitor cells (OPCs), glial precursors and astrocytes can also be transformed under certain experimental conditions and are present in the SVZ. Furthermore, it should be noted that there is not a single type of NSC; this is a general term that encompasses diverse cell types with different transcriptional and epigenetic profiles, spatial and temporal identities, and associated differentiation biases (Obernier and Alvarez-Buylla, 2019). How these distinct ‘flavours’ of an NSC relate to the features of the resulting tumour or their differentiation behaviour remains a major area of investigation. Another consideration is the cell-cycle status. A continuum of distinct cell-cycle states (dormant, primed quiescent, and activated) have been found in single-cell analysis of the mouse SVZ (Llorens-Bobadilla et al., 2015). However, the range of quiescent states and their relationship to normal differentiation programmes remains unknown.

Regardless of their origin, it is clear that GBM cells frequently express a range of NSC markers, many of which also have key functional roles: for example, neurodevelopmental transcription factors (TFs), e.g. SOX, HOX, bHLH, ZF-TFs and FOX family members. These have emerged as key effectors of the unconstrained self-renewal of GBM stem cells (GSCs) that drives the disease (Gallo et al., 2013; Bulstrode et al., 2017; Lu et al., 2016; Singh et al., 2017; Suvà et al., 2014). Induction of their expression may be one of the key outputs of the receptor tyrosine kinase signalling pathways (Liu et al., 2015). Elevated activity of these master regulatory and reprogramming factors may therefore explain the limited terminal differentiation capacity of GSCs (Carén et al., 2015). They may be locked into a perpetual cycle of self-renewal (Bulstrode et al., 2017; Suva et al., 2013).

Comparison of single-cell profiling data suggests that GSCs have transcriptional profiles similar to those of the outer SVZ/radial glia foetal progenitors, which are a specific subset of amplifying progenitors in the developing human cortex (Pollen et al., 2015; Patel et al., 2014). Transcriptional resetting to a foetal-like state may therefore be a feature of GSCs. Stemness-associated neurodevelopmental pathways and transcriptional/epigenetic programmes are therefore an area ripe for identification of therapeutic targets, defining new biological vulnerabilities that might not be uncovered through genome sequencing alone (Mack et al., 2015).

GBM arises in the most complex organ in our bodies. The elaborate tumour microenvironment (TME) influences tumour cell fate in many ways. NSCs exist in a range of proliferative and non-cycling/quiescent states (Patel et al., 2014), and local niches regulate this balance (Hambardzumyan and Bergers, 2015). The acquisition of a quiescent state by GSCs may explain resistance to cytotoxic and anti-mitotic agents (Bao et al., 2006; Chen et al., 2012a). The vasculature in GBM forms a key niche that supports brain-tumour stem-cell self-renewal (Gilbertson and Rich, 2007) and mediates signals that impose a quiescent state (Ottone et al., 2014). The vasculature in the tumour margin also comprises endothelial cells with specialised tight junctions, pericytes and astrocyte processes; this is a selective barrier, termed the blood-brain barrier (BBB). This protects the brain, but limits delivery of drugs or biological therapeutics to the infiltrative tumour cells. Although the BBB is disrupted in the main tumour mass, cells within the infiltrative margin, which is responsible for tumour regrowth, often infiltrate widely into macroscopically normal surrounding tissue.

We still have a limited understanding of how the microenvironment shapes cell quiescence, proliferation, differentiation and infiltration. Do subsets of cells in the tumour’s infiltrative margin harbour distinct genetic or epigenetic disruptions (Piccirillo et al., 2015)? How can they thrive and propagate in the absence of paracrine growth factors or niche signals? Do they exist in different states when infiltrating via endothelial versus white-matter routes? Is the balance of these fates determined mainly by certain oncogenic drivers?

Immunotherapy with checkpoint inhibitors has not proven straightforward for GBM, although encouraging results have been reported recently (Ito et al., 2019). There is evidence for the presence of T cells, macrophages and immune cytokines in the GBM TME, and a glymphatic system exists – a peri-vascular network dependent on glia with a pseudo-lymphatic function (Plog and Nedergaard, 2018). Much research is also needed to understand how this tumour immune microenvironment operates in the context of GBM and how it can be exploited therapeutically.

In summary, GBM models must be suitable to study diverse processes, including: neurodevelopmental transcriptional and epigenetic programmes; the balance between dormancy, quiescence, proliferation and differentiation; infiltration via endothelial, white-matter or other routes; the BBB; immune regulation; mechanosignalling; and responses to standard of care (radio- and chemotherapy).

Current models encompass five major categories that we discuss below: (1) GBM cell lines and primary cultures/explants (primary-tumour derived); (2) *in vitro* engineered tumour-initiating cells (e.g. transformed cultured NSCs); (3) *ex vivo*, brain/tumour slice culture models; (4) *in vivo* mouse transplantation of tumour-initiating cells; (5) genetically engineered mouse models (GEMMs), often referred to as *de novo*, or autochthonous, models (via breeding strategies and/or delivery of somatic mutations).

## *In vitro* models: an abundance of choice

*In vitro* models are tractable and cost effective. They enable a reductionist approach that is best suited to the dissection of cell-intrinsic properties using biochemical, cell-biological and reverse-genetics approaches. This views the cultured cells as autonomous renegade cells, with features more akin to a microorganism in terms of growth and self-sufficiency. Researchers can generate large populations, which simplifies experimental approaches such as chemical/genetic screens, transcriptomics and proteomics. Clonal experiments or other single-cell analyses are straightforward, providing rigorous information without the potentially confounding complexity of diverse extrinsic signals. A major risk of working with cultured cells is that they may diverge, genetically or epigenetically, to the point of being non-relevant to the human disease. Thus, to validate findings, careful consideration and controls must be in place to ensure the disease relevance of any new findings, and *in vitro* discoveries always need to be complemented with *in vivo* models.

There is a choice of working with established ‘classic’ cell lines versus more recently developed patient-derived models grown in NSC culture conditions. Widely used ‘classic’ cell lines, such as U87MG, U251 and T98G, are grown in serum-supplemented media, but these culture conditions promote astrocytic differentiation. Inadvertently, investigators have therefore forced cells into a differentiated astrocytic state, with transcriptional and epigenetic programmes that do not reflect the neural stem/progenitor pathways that underlie GSCs (Lee et al., 2006). The tumours that develop upon xenotransplantation of such serum-grown cell lines do not resemble GBM (Lee et al., 2006). This casts doubt on the value of any study that has relied on these models. Furthermore*,* recent research has shown that the U87MG cells distributed by ATCC – one of the most popular cell lines (Pontén and Macintyre, 1968) – was in fact likely switched with another cell line, as it does not match the original Uppsala stocks (Allen et al., 2016). Although they are popular, our view is that classic cell lines have limited utility, either for reductionist mechanistic studies or for preclinical testing of agents. The field must move away from these, as also advocated by Westermark et al. and Xie et al. (Allen et al., 2016; Xie et al., 2015).

Primary-culture conditions to expand neural stem and progenitor cells from the adult and developing CNS were first reported around 1990 (Temple, 1989; Reynolds and Weiss, 1992; Ray et al., 1993). These studies described long-term culture of mouse NSCs from both foetal brain tissue and from the adult SVZ using suspension culture, as neurospheres (Reynolds and Weiss, 1992). The key features of this approach were a lack of serum and presence of EGF in the culture media.

Patient-derived primary GBM cells cultured under similar conditions can be sustained long term, either in suspension or adherent (on laminin) culture (Galli et al., 2004; Hemmati et al., 2003; Pollard et al., 2009; Singh et al., 2003). These retain the genetics and transcriptional state of the parental tumour, unlike the serum-grown ‘classic’ cell lines (Lee et al., 2006). The GSCs that emerge in these culture conditions also more faithfully recapitulate the features of primary tumours when transplanted into rodent brains, even after many passages. Thus, they provide a human, disease-relevant, *in vitro* model with stem-cell-like features. Genetic disruptions in the parental tumour are well retained following long-term culture, as well as within the resulting xenografts – including the previously mentioned variable extrachromosomal elements carrying oncogenes (deCarvalho et al., 2018). Repositories of such cells are now being developed to make these models accessible to the research community (www.gcgr.org.uk; Xie et al., 2015). It is important to reiterate that these cultures are established without any genetic manipulations or cell sorting: the culture conditions ‘capture’ the GSC state, which enables experiments that encompass some degree of genetic diversity of the original tumour.

NSCs and GSCs were originally expanded in suspension culture as neurospheres. However, growth in suspension culture is not a defining feature of stem cells and is not essential for their long-term expansion (Conti et al., 2005; Sun et al., 2008). Working with cells in adherent monolayers offers many experimental advantages, particularly with regards to culture homogeneity, imaging approaches, clonal propagation/picking, screening and quantitation (Conti et al., 2005), thereby overcoming some inherent limitations of working with suspension culture (Pastrana et al., 2011). Our own group and others have also reported a much greater success in deriving new GBM cell lines when using adherent culture, with >90% success for IDH wild-type GBM (Pollard et al., 2009; Xie et al., 2015).

In 2014, Lancaster et al., building upon previous ES-cell self-organisation studies of the Sasai lab (Kadoshima et al., 2013), described a method for the generation of neural tissue from human pluripotent stem cells (hPSCs) with some of the organised features of the developing cortex (Lancaster et al., 2013). These have been termed ‘organoids’ to highlight their similarities to existing organoid systems defined for endodermal stem cell cultures, like the use of Matrigel in suspension (Huch et al., 2017; Tuveson and Clevers, 2019). Organoid culture paradigms enable the *ex vivo* growth of primary GBM specimens to a large size (Hubert et al., 2016). This allows modelling of the necrotic and hypoxic features of human tumours, alongside the corresponding greater range of quiescent, proliferative and differentiating cell states (Hubert et al., 2016). However, generation of cerebral organoids is highly variable and takes months of culture. Choosing between growing cells in an adherent monolayer versus suspension culture, either as spheres or organoids, is therefore influenced by whether working with purer populations and homogeneity is essential (reductionist questions), or whether researchers need the complexity and heterogeneity (necrosis, quiescence/proliferation and differentiation) that is triggered in suspension culture and is more reminiscent of the patient tumour.

In summary, GBM researchers are in the fortunate position of being able to expand primary patient cells routinely from fresh patient tumours, and classic cell lines are no longer required. Cells can be grown as pure adherent cultures or in suspension or organoid culture conditions to recreate more complex 3D models. Normal neural stem and progenitor cells can also be isolated and expanded in culture or generated from PSCs (i.e. iPSCs or hESCs). Arguably, for no other human cancer are we in such a favourable position in the choice and flexibility of mouse and human *in vitro* models.

## Engineering GBM *in vitro*

GSCs display many features of foetal NSCs, such as many of the molecular markers that are expressed within a specific progenitor cell termed the outer SVZ radial glia (Pollen et al., 2015). Human foetal NSCs can be easily derived, and retain a diploid karyotype and differentiation capacity over multiple passages (Sun et al., 2008). Comparison of GSCs to ‘normal’, non-malignant human foetal NSCs has provided insights into the differential molecular programmes underlying acquisition of the malignant phenotype. Adherent human NSCs can also be obtained via *in vitro* differentiation of hPSCs (Conti et al., 2005), although primary human foetal NSCs arguably provide a more reliable starting source for comparison to GBM.

NSCs can be expanded *in vitro* and differentiated into astroglial and oligodendrocyte progeny. These NSCs, and perhaps also their immature precursor-cell descendants, are a likely cell of origin for GBM and can be readily genetically manipulated in culture. An obvious experimental strategy is therefore to model GBM by engineering driver mutations stepwise and in combinations *in vitro* and subsequently transplant the cells *in vivo* (see below).

A range of standard molecular biology approaches have been used to deliver oncogenes and short hairpin RNAs (shRNAs), including plasmid transfection and lentiviral or retroviral transduction. Bachoo et al. showed that postnatal primary cortical astrocytes and NSCs from *cdkn2a* (encoding Ink4a and ARF)-null mice can be transformed *in vitro* using retrovirus to induce constitutive expression of the GBM-associated oncogenic protein EGFRvIII (Bachoo et al., 2002). The transduced primary cortical astrocytes and NSCs formed tumours when transplanted into the brains of immunocompromised mice.

NSCs derived from differentiating PSCs have also been transformed into glioma-initiating cells. Funato et al. derived neural progenitor cells from human ESCs to model DIPG *in vitro* and to study the effects of the histone H3.3K27M mutation on cellular growth kinetics and tumorigenicity (Funato et al., 2014). They used lentiviral transduction to introduce activated PDGFRA and wild-type or mutant H3.3 along with an shRNA against *TP53*. Instead of viral transduction, researchers can also use transposases (e.g. the PiggyBac system) for stable random integration of oncogene expression cassettes (Ding et al., 2005).

### Precision engineering: genome editing with CRISPR

**Fig. 2.**
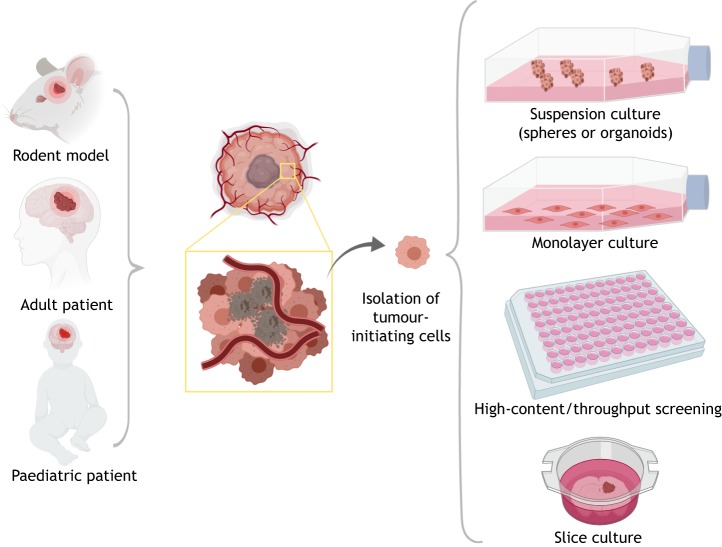
**Sources of GBM tumour cells and their capture *in vitro*.** Tumour tissue and tumour cell populations can be obtained from rodent models (see Fig. 3) or patients (adult or paediatric). Tumour-initiating cells can be maintained in culture using neural-stem-cell culture conditions (serum-free media with growth factors EGF and FGF2). These can be expanded in suspension as spheres or organoids, or in an adherent monolayer. Clonal cell lines can be obtained, and cells plated in microtiter plates for arrayed genetic or chemical screens. Cells and tumour explants can also be engrafted on brain slice cultures to model tumour-host interactions.

The emergence of CRISPR/Cas9 technology has transformed many areas of biology, including cancer research (Hsu et al., 2014; Wright et al., 2016). Genome editing with CRISPR/Cas9 now enables not only genetic knockout of tumour suppressors (preferred over RNAi-mediated knockdown), but also a range of more complex and precise genetic changes such as knock-ins or engineering of complex alleles (Fig. 2). These CRISPR-based techniques have also opened up possibilities for new genetic screening approaches, both *in vitro* (Toledo et al., 2015) and *in vivo* (Chow et al., 2017), and, importantly, allow researchers to generate isogenic cell line pairs for precisely controlled experimentation.

**Fig. 3.**
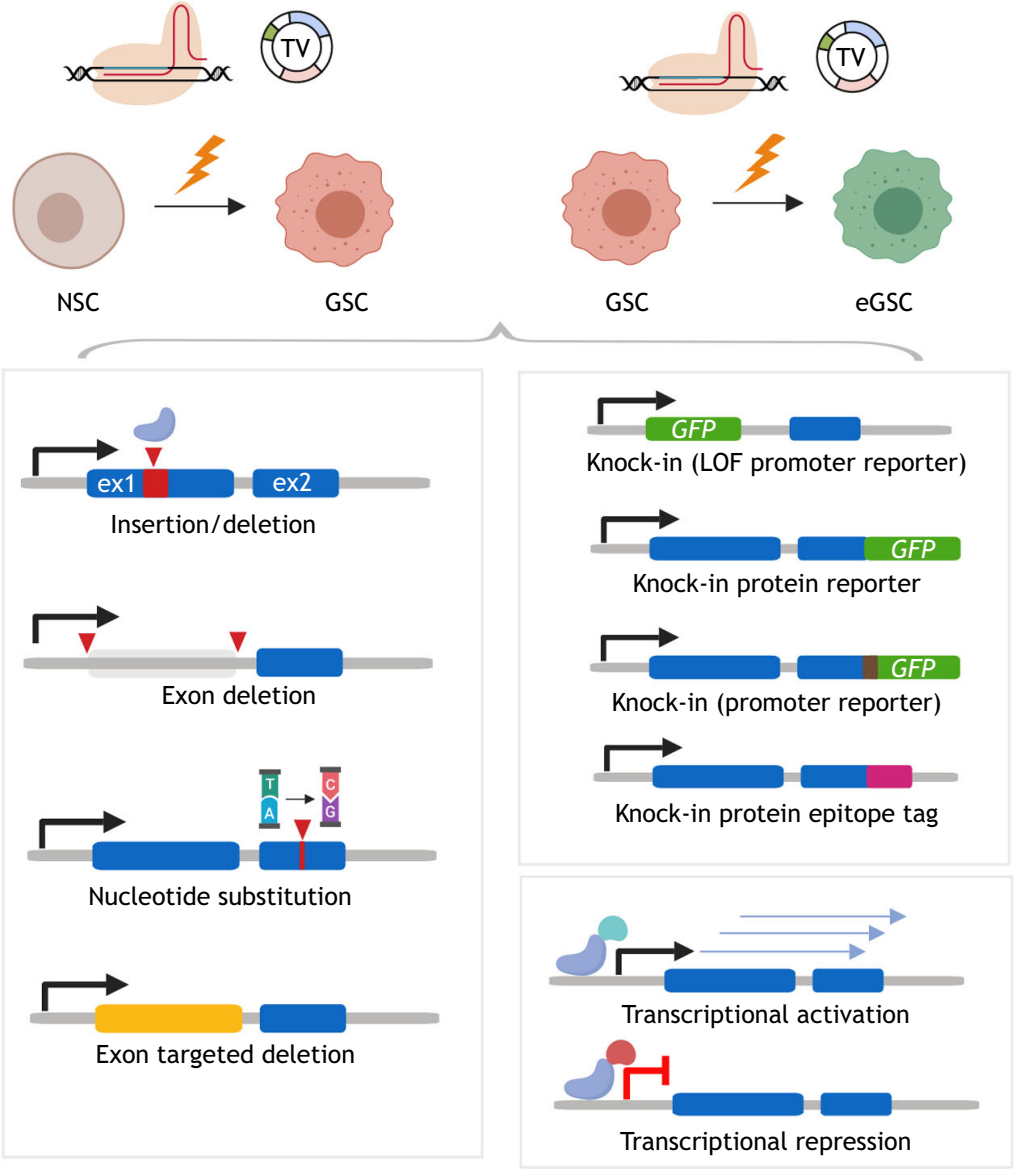
**Engineering NSCs and GSCs with CRISPR-based genome editing.** A variety of different genetic or epigenetic manipulations can be introduced using CRISPR/Cas-assisted gene engineering, either mutations (bottom left) or knock-in alleles (bottom right). ex1/2, exon 1/2; NSC, neural stem cell; GSC, glioblastoma stem cell; eGSC, engineered glioblastoma stem cell; GFP, green fluorescent protein; LOF, loss of function; TV, targeting vector.

CRISPR/Cas9-induced cuts to genomic DNA can be repaired by cellular mechanisms that result in efficient gene disruption with knockouts via formation of insertion/deletion mutations. They can also be repaired by homologous recombination combined with gene targeting to introduce specific point mutations or more sophisticated modifications, such as the knock-in of epitope tags, protein fusions or reporters (Dewari et al., 2018). Bressan et al. demonstrated that CRISPR/Cas9-mediated gene targeting by homologous recombination is efficient in mouse and human NSCs (Bressan et al., 2017). In the coming years, we will see them deployed for lineage tracing (CreERT2 knock-in), label-retaining assays for quiescence (H2B-GFP pulse-chase experiments), conditional alleles (*loxP* or *frt*-based recombination), knock-in of degrons [e.g SMAsh-tag (Chung et al., 2015)] and engineering of more complex chromosomal structural changes (Choi and Meyerson, 2014).

Recently, independent groups have used CRISPR/Cas9 technology in human organoid culture systems to engineer oncogene constructs or disrupt tumour suppressors such as the *TP53* locus (Bian et al., 2018; Ogawa et al., 2018). Cells isolated from these organoid tumours bear the molecular signature of mesenchymal GBM samples, express markers of heterogeneous cell types and can be transplanted into mice, where they form tumours (Ogawa et al., 2018).

Such isogenic panels of engineered transformed cells and their parental controls provide the critical models that can improve target identification and validation in drug discovery efforts. This overcomes the obstacle of genetic variation in mechanistic studies. Rigorous functional genetic studies probe the genes and pathways regulating key facets of GBM biology and can address some of the common pitfalls in preclinical cancer-target validation studies (Kaelin, 2017).

## *In vivo* modelling: transplantation and genetically engineered models

Despite their many advantages, *in vitro* cellular models have limited scope for exploration of extrinsic signals regulating GBM stem-cell fate, such as tumour-host interactions and immune control. *Ex vivo* modelling approaches include organotypic brain slice cultures. These are useful for bridging the gap between *in vitro* cell culture studies and the *in vivo* animal studies, and have been extensively used in neuroscience to explore neuronal electrical activity (Humpel, 2015). Slice culture methods offer opportunities for imaging and tracking cell responses with great precision over microanatomical location in the correct brain-tissue architecture, such as GBM cell interactions with the SVZ niche (Marqués-Torrejón et al., 2017). However, whole-animal models undoubtedly provide the key disease-relevant models for GBM.

**Fig. 4.**
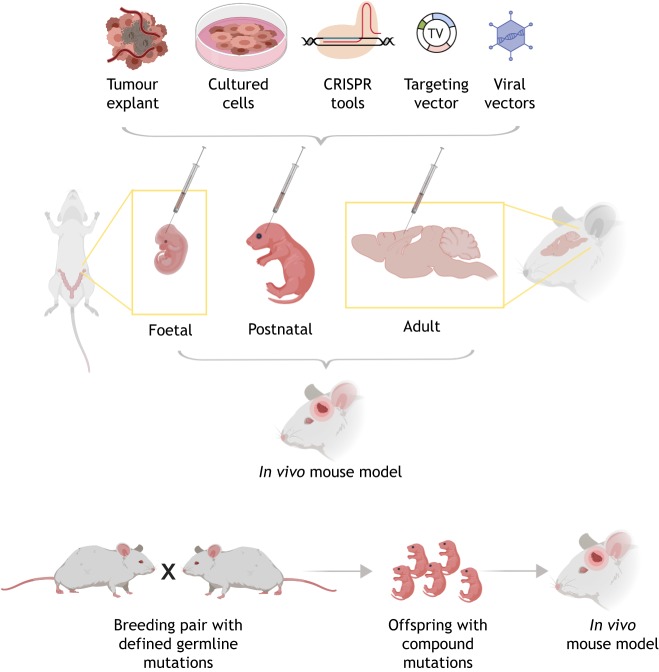
***In vivo* mouse models can be generated through transplantation of cells or tumour tissues, or through engineered driver mutations (by breeding or somatic mutation).** (Top panel) Shown are foetal, postnatal and adult brain injections of either tumour explants, cells, CRISPR ribonucleoproteins, plasmids or viral vectors (viral delivery of genetic material). Bottom panel: *in vivo* mouse models can also be generated by breeding animals that carry germline mutations.

Mice are by far the most experimentally accessible mammalian model. This is primarily due to their ease of genetic manipulation, short breeding times, and shared organ systems and physiology. Transplantation of tumour-initiating cells into mice provides a relatively low-cost model for the rapid interrogation of tumour biology and for identifying therapeutic vulnerabilities. These can be either transformed/engineered cells, or cancer cells from primary tumours. However, the downside is the disruption of tumour tissue architecture and potential selection events that occur within the transplantation procedure, and so these approaches are complemented by autochthonous models in which *de novo* tumours are formed by using genetic approaches. Mutations can be either introduced via the germ line and breeding strategies, or through somatic cell mutation (Fig. 3). These complementary strategies for studying tumours *in vivo* (Fig. 4) are discussed in detail below.

### Transplantation of tumour-initiating cells

Transplants can be allografts, in which the implanted cancer cells are from the same species as the recipient, e.g. mouse into mouse, or xenografts, where implanted cells are from a different species, e.g. human into mouse. The resulting grafts can be orthotopic – i.e. transplanted intracranially, typically into the brain with stereotactic surgery – or heterotopic, most typically subcutaneous. The former is clearly more attractive, as it provides the correct tissue/organ context. Subcutaneous injection has been widely used because it is easy technically and therefore enables larger throughput, but cannot be used to explore brain infiltrative behaviour and lacks appropriate brain microenvironments (Liu et al., 2015). Subcutaneous transplants are hence undesirable; investigators should avoid using this approach if possible.

An advantage of orthotopic xenografts is the precise control of spatial and temporal tumour initiation. Large cohorts of tumour-bearing mice can therefore be generated with consistent tumour sizes and sites. Monitoring of the transplanted tumour cells using bioluminescence *in vivo*, which requires stable expression of a luciferase cassette in the transplanted cells, is now widely used and enables longitudinal tracking of tumour growth. The downsides are that this approach typically requires large numbers of cells for injection, and there is limited ability to control events during engraftment and seeding steps. Also, the injection procedure itself inevitably creates an injury, thereby disrupting normal tissue architecture and physiology.

Transplantation into syngeneic hosts has the advantage of modelling immune interactions. Originally, GBM cell lines were generated from carcinogen-induced rodent gliomas or from transgenic mice, cultured, and transplanted into syngeneic hosts. This approach was used to generate the GL261 cell line, which is often used for immune studies (Akbasak et al., 1991). However, GL261 has genetically drifted and does not model an authentic GBM-like tumour (Szatmári et al., 2006). Histologically they do not match GBM, and they have accrued mutations, such as in *KRAS*, a mutant allele that is not associated with GBM. With the advent of CRISPR technology, as discussed above, researchers can achieve specific genetic alterations through stepwise engineering of adult mouse NSCs, rendering them tumorigenic when orthotopically transplanted into the brains of isogenic mice with a fully functional immune system. So, a new range of models will soon emerge, enabling studies of GBM immune regulation.

Human cell lines or patient-derived cells require transplantation into immunocompromised mice to prevent immune rejection. Direct implantation of freshly isolated tumour cells or tissue fragments, without intervening cell culture steps, has been used to create patient-derived orthotopic xenograft (PDOX) models. This has the advantage of capturing genetic diversity, as well as aspects of the TME, e.g. vessels, the extracellular matrix and likely some immune regulators, providing the most direct attempt to capture disease-relevant features of the tumours without any *in vitro* selection. Maintenance of PDOX models is costly and labour intensive, limiting access to a few institutions. These models also cannot sidestep the inherent problem of selection and drift that inevitably occurs as the tumours are propagated through mice – both for distinct subpopulations of tumour cells and for the loss of human TME as murine stroma takes over. Ben-David and colleagues assessed copy number alterations (CNAs) in patient-derived xenografts from multiple cancer types across serial *in vivo* passages and found a striking rates of CNA (Ben-David et al., 2017). So, in prolonged *in vivo* culture, direct patient xenografts may actually perform no better than GSC cultures expanded *in vitro* prior to transplantation (deCarvalho et al., 2018). GSCs have the advantage that cells can be fully characterised, archived and distributed to the community.

### Genetically engineered mouse models: germ-line and somatic mutation

Before the sophisticated modern tools of molecular biology emerged, researchers used chemical mutagenesis, e.g. with N-ethyl-nitrosurea (ENU), to develop glioma models (Schiffer et al., 1978). Such tumours harbour mutations found in human GBM, display genetic heterogeneity, and arise within a disease-relevant microenvironment in an immunocompetent host. However, the efficiency and reproducibility of tumour formation is low. Polyclonal origins and lack of control of the specific genetic drivers are also an issue. For these reasons, GEMMs have become the favoured option.

GEMMs are created by introducing defined genetic alterations in the germline and using breeding strategies that generate compound mutants with alterations in both oncogenes and tumour suppressors. Such autochthonous models can provide valuable insights into early initiation events. Inevitably, mutations in some of the relevant genes are early lethal and therefore must be engineered using conditional approaches (e.g. Cre-*loxP* recombination strategies). CreERT2 driver alleles result in tissue-restricted and temporally controlled tumour-suppressor deletion through Cre recombinase induction with tamoxifen.

A key mouse breeding model for primary GBM was reported by the Parada group by combining *Trp53* loss and conditional loss of *Nf1* (Zhu et al., 2005)*.* This important study demonstrated the functional importance of *Nf1* loss in driving malignant astrocytoma. In fact, this preceded the realisation that *Nf1* loss is a recurrent driver in GBMs (Cancer Genome Atlas Research Network, 2008). Using this fully penetrant mouse model and combining it with various Cre drivers, this group has been able to explore, using elegant mouse genetics, the candidate cell of origin for GBM (Alcantara Llaguno et al., 2015, 2016, 2009, 2019) and the importance of the quiescent GBM stem-cell-like population in driving relapse (Chen et al., 2012a). NG2-CreER mice demonstrated that the proneural subtype was likely derived from OPCs, whereas other GBM subtypes resembled tumours generated in *Nes-CreER* mice, suggesting a CNS progenitor cell of origin (Alcantara Llaguno et al., 2015). Similar studies using autochothonous models and *de novo* tumour formation also suggested a lower barrier to malignant transformation in the NSCs than in astrocytes (Chow et al., 2011). For *IDH*-mutant GBM, Bardella et al. used conditional expression of the *IDH1*^R132H^ allele in the adult SVZ to model the early events of gliomagenesis (Bardella et al., 2016).

A related approach is to initiate tumours by delivery of a Cre-expressing virus, thus spatially restricting mutations to specific brain regions (e.g. cortex or SVZ). This has provided evidence that, following ablation of the key tumour suppressors *Rb*, *Trp53* or *Pten*, SVZ NSCs are more easily transformed than parenchymal differentiated astrocytes (Jacques et al., 2010).

Viral delivery can also be used to introduce GBM oncogenes *in vivo*. A lentivirus-based delivery system for HRas and AKT overexpression also indicated that cells within the NSC-containing regions were more easily transformed than cells in other brain regions (Marumoto et al., 2009). A popular approach has been the RCAS-TVA system. Cells producing TVA, the receptor for subgroup A avian leukosis viruses, are susceptible to infection with replication-competent avian sarcoma-leukosis virus long terminal repeat with splice acceptor (RCAS) viral vectors. RCAS-TVA has contributed to our understanding of the potential cell(s) of origin of GBM (Holland et al., 2000). Holland et al. developed transgenic mouse lines expressing the TVA in *Nes-* or *Gfap-*expressing cells, presumed to be progenitor cells and differentiated astrocytes, respectively, and bred these with *Cdkn2a*-knockout mice (Holland et al., 1998). *Nes*-TVA mice were more susceptible to tumour formation than *Gfap*-TVA (Holland et al., 2000). However, endogenous human and mouse NSCs with self-renewal and differentiation capacity also express GFAP (Doetsch et al., 1999), and so this marker alone does not distinguish differentiated astrocyte populations. Jiang et al. also used RCAS with lineage-restricted promoters and confirmed a significant impact of differentiation state on tumour aggressiveness, with more restricted progenitors being less malignant (Jiang et al., 2017). Recent research demonstrated the utility of combining CRISPR/Cas9, as this system can deliver oncogenes and/or also induce loss-of-function mutations in tumour suppressors (Oldrini et al., 2018). A limitation of the RCAS-TVA system is the need to breed specific TVA-expressing mouse strains. Moreover, there are viral cargo limitations (maximum 2.5 kb), which poses some restriction (e.g. the *EGFRvIII* oncogene is 2.8 kb long).

A further constraint of all of autochthonous models – either those developed via breeding or somatic mutation – is the possibility for polyclonal tumour initiation. For the models generated by the Parada group (discussed above; Chen et al., 2012a), this was indeed an issue, as survival data were complicated by the emergence of spontaneous tumours in the hindbrain. So, although providing a useful tool to generate highly penetrant autochthonous tumours, this approach is likely to become superseded by CRISPR- and PiggyBac-based approaches that can deliver combinations of oncogenes and tumour suppressors in multiplex, directly *in vivo*, and with high enough efficiency for tumour formation (Pathania et al., 2017). These plasmid-based approaches do not require mouse breeding or virus production, and enable the delivery of larger cargo sizes.

## Future prospects

What are the potential improvements in GBM models in coming years? The lack of a human immune system is a limitation for patient-derived xenografts. Given the importance of cancer immunotherapy in the clinic, immunocompetent models are urgently needed to understand how to overcome the immunosuppressive mechanisms in GBM. Strategies to develop mice with a humanised immune system are emerging (Billerbeck et al., 2011; Mahne et al., 2017; Shultz et al., 2012).

CRISPR-based approaches could be used to engineer multiplex inducible GBM drivers in human NSCs, which could then be engrafted into a foetal mouse brain, enabling the generation of a *de novo* chimeric tumour. Also, with improvements in iPSC culture and differentiation protocols, it will become possible to produce homogeneous populations of isogenic primary human cells (e.g. microglia, macrophages and endothelial cells). These could be studied in co-culture with tumour cells *in vitro* or following transplantation to explore host-tumour cell interactions. iPSC technology combined with genome editing can therefore create complex *ex vivo* models that will likely be helpful in the triage of compounds in small-molecule drug discovery programmes.

Advances in genome editing technologies now mean that, to some extent, all animals have the potential to become genetically manipulable, and in the future this will drive a new range of large animal models to complement and support mouse and human studies. Immunocompromised strains of the Yucatan minipig have been used as a host for human-cell-line xenografts (Khoshnevis et al., 2017). The size and gyrencephalic structure of the porcine brain, along with a BBB physiology similar to that in humans, makes it a more comparable model to the human brain than are rodent brains. Dogs also provide a useful model of GBM as the disease arises in them spontaneously, generating, in an immunocompetent host, similar heterogeneous infiltrative tumours to those found in humans (Koehler et al., 2018). Preclinical testing of new therapeutics – whether small molecule, biologics, or gene or cell therapy – should therefore have a much greater quality and diversity of available models. This will underpin better-quality clinical trials based upon strong scientific evidence. It is also clear that testing of new therapeutics in models needs to incorporate the current standard of care to ensure therapies are tested in a manner that will closely relate to existing clinical care and clinical trials design; i.e. treating the mouse in ‘mouse hospitals’ with surgical debulking, radiotherapy (using small-animal radiation research platforms) and temozolomide regimes. This will be expensive and logistically challenging; even more so when one considers that these control tests would ideally be performed in large animal models.

## Conclusions

Our knowledge of the origins and molecular programmes underpinning GBM has steadily expanded. GBMs are driven by simultaneous disruptions to ‘classic’ cancer signalling pathways that operate in the context of a neural stem or progenitor cell state. These mutated pathways cannot easily be blocked or reversed with targeted therapies due to pathway redundancy and extensive intra-tumoural heterogeneity. New approaches will be needed that focus on functional studies and deep understanding of the tumour biology. No single approach will suffice. Fortunately for GBM, we are witnessing the emergence of a range of high-quality and complementary mammalian and human models. The community will need to share these and associated tools to stimulate a new era of greater cross-collaboration between the fundamental research, translational and drug discovery effort, and clinical studies. We are optimistic that the long-awaited new discoveries, new validated targets and new therapeutic strategies will emerge.

## References

[DMM040386C1] Akbasak A., Oldfield E. H. and Saris S. C. (1991). Expression and modulation of major histocompatibility antigens on murine primary brain tumor in vitro. *J. Neurosurg.* 75, 922-929. 10.3171/jns.1991.75.6.0922 1941122

[DMM040386C2] Alcantara Llaguno S., Chen J., Kwon C.-H., Jackson E. L., Li Y., Burns D. K., Alvarez-Buylla A. and Parada L. F. (2009). Malignant astrocytomas originate from neural stem/progenitor cells in a somatic tumor suppressor mouse model. *Cancer Cell* 15, 45-56. 10.1016/j.ccr.2008.12.006 19111880 PMC2650425

[DMM040386C3] Alcantara Llaguno S. R., Wang Z., Sun D., Chen J., Xu J., Kim E., Hatanpaa K. J., Raisanen J. M., Burns D. K., Johnson J. E. et al. (2015). Adult lineage-restricted CNS progenitors specify distinct glioblastoma subtypes. *Cancer Cell* 28, 429-440. 10.1016/j.ccell.2015.09.007 26461091 PMC4607935

[DMM040386C4] Alcantara Llaguno S. R., Xie X. and Parada L. F. (2016). Cell of origin and cancer stem cells in tumor suppressor mouse models of glioblastoma. *Cold Spring Harb. Symp. Quant. Biol.* 81, 31-36. 10.1101/sqb.2016.81.030973 27815542 PMC6353557

[DMM040386C5] Alcantara Llaguno S., Sun D., Pedraza A. M., Vera E., Wang Z., Burns D. K. and Parada L. F. (2019). Cell-of-origin susceptibility to glioblastoma formation declines with neural lineage restriction. *Nat. Neurosci.* 353, 811 10.1038/s41593-018-0333-8 PMC6594191 30778149

[DMM040386C6] Aldape K., Brindle K. M., Chesler L., Chopra R., Gajjar A., Gilbert M. R., Gottardo N., Gutmann D. H., Hargrave D., Holland E. C. et al. (2019). Challenges to curing primary brain tumours. *Nat. Rev. Clin. Oncol.* 16, 509-520. 10.1038/s41571-019-0177-5 30733593 PMC6650350

[DMM040386C7] Allen M., Bjerke M., Edlund H., Nelander S. and Westermark B. (2016). Origin of the U87MG glioma cell line: Good news and bad news. *Sci. Transl. Med.* 8, 354re3 10.1126/scitranslmed.aaf6853 27582061

[DMM040386C8] Bachoo R. M., Maher E. A., Ligon K. L., Sharpless N. E., Chan S. S., You M. J., Tang Y., DeFrances J., Stover E., Weissleder R. et al. (2002). Epidermal growth factor receptor and Ink4a/Arf: convergent mechanisms governing terminal differentiation and transformation along the neural stem cell to astrocyte axis. *Cancer Cell* 1, 269-277. 10.1016/S1535-6108(02)00046-6 12086863

[DMM040386C9] Bao S., Wu Q., McLendon R. E., Hao Y., Shi Q., Hjelmeland A. B., Dewhirst M. W., Bigner D. D. and Rich J. N. (2006). Glioma stem cells promote radioresistance by preferential activation of the DNA damage response. *Nature* 444, 756-760. 10.1038/nature05236 17051156

[DMM040386C10] Bardella C., Al-Dalahmah O., Krell D., Brazauskas P., Al-Qahtani K., Tomkova M., Adam J., Serres S., Lockstone H., Freeman-Mills L. et al. (2016). Expression of Idh1(R132H) in the murine subventricular zone stem cell niche recapitulates features of early gliomagenesis. *Cancer Cell* 30, 578-594. 10.1016/j.ccell.2016.08.017 27693047 PMC5064912

[DMM040386C11] Ben-David U., Ha G., Tseng Y.-Y., Greenwald N. F., Oh C., Shih J., McFarland J. M., Wong B., Boehm J. S., Beroukhim R. et al. (2017). Patient-derived xenografts undergo mouse-specific tumor evolution. *Nat. Genet.* 49, 1567-1575. 10.1038/ng.3967 28991255 PMC5659952

[DMM040386C12] Bian S., Repic M., Guo Z., Kavirayani A., Burkard T., Bagley J. A., Krauditsch C. and Knoblich J. A. (2018). Genetically engineered cerebral organoids model brain tumor formation. *Nature Publishing Group* 15, 631-639. 10.1038/s41592-018-0070-7 PMC6071863 30038414

[DMM040386C13] Billerbeck E., Barry W. T., Mu K., Dorner M., Rice C. M. and Ploss A. (2011). Development of human CD4+FoxP3+ regulatory T cells in human stem cell factor-, granulocyte-macrophage colony-stimulating factor-, and interleukin-3-expressing NOD-SCID IL2Rγ(null) humanized mice. *Blood* 117, 3076-3086. 10.1182/blood-2010-08-301507 21252091 PMC3062310

[DMM040386C14] Brennan C. W., Brennan C. W., Verhaak R. G. W., Verhaak R. G. W., McKenna A., Campos B., Campos B., Noushmehr H., Noushmehr H., Salama S. R. et al. (2013). The somatic genomic landscape of glioblastoma. *Cell* 155, 462-477. 10.1016/j.cell.2013.09.034 24120142 PMC3910500

[DMM040386C117] Bressan R., Dewari P., Kalantzaki M., Gangoso E., Matjusaitis M., Garcia-Diaz C., Blin C., Grant V., Bulstrode H., Gogolok S. et al. (2017). Efficient CRISPR/Cas9-assisted gene targeting enables rapid and precise genetic manipulation of mammalian neural stem cells. *Development* 144, 635-648. 10.1242/dev.140855 28096221 PMC5312033

[DMM040386C15] Bulstrode H., Johnstone E., Marqués-Torrejón M. Á., Ferguson K. M., Bressan R. B., Blin C., Grant V., Gogolok S., Gangoso E., Gagrica S. et al. (2017). Elevated FOXG1 and SOX2 in glioblastoma enforces neural stem cell identity through transcriptional control of cell cycle and epigenetic regulators. *Genes Dev.* 31, 757-773. 10.1101/gad.293027.116 28465359 PMC5435889

[DMM040386C16] Burnet N. G., Jefferies S. J., Benson R. J., Hunt D. P. and Treasure F. P. (2005). Years of life lost (YLL) from cancer is an important measure of population burden--and should be considered when allocating research funds. *Br. J. Cancer* 92, 241-245. 10.1038/sj.bjc.6602321 15655548 PMC2361853

[DMM040386C17] Cancer Genome Atlas Research Network. (2008). Comprehensive genomic characterization defines human glioblastoma genes and core pathways. *Nature* 455, 1061-1068. 10.1038/nature07385 18772890 PMC2671642

[DMM040386C18] Capper D., Jones D. T. W., Sill M., Hovestadt V., Schrimpf D., Sturm D., Koelsche C., Sahm F., Chavez L., Reuss D. E. et al. (2018). DNA methylation-based classification of central nervous system tumours. *Nature* 555, 469-474. 10.1038/nature26000 29539639 PMC6093218

[DMM040386C19] Carén H., Stricker S. H., Bulstrode H., Gagrica S., Johnstone E., Bartlett T. E., Feber A., Wilson G., Teschendorff A. E., Bertone P. et al. (2015). Glioblastoma stem cells respond to differentiation cues but fail to undergo commitment and terminal cell-cycle arrest. *Stem Cell Rep.* 5, 829-842. 10.1016/j.stemcr.2015.09.014 PMC4649264 26607953

[DMM040386C20] Chen J., Li Y., Yu T.-S., McKay R. M., Burns D. K., Kernie S. G. and Parada L. F. (2012a). A restricted cell population propagates glioblastoma growth after chemotherapy. *Nature* 488, 522-526. 10.1038/nature11287 22854781 PMC3427400

[DMM040386C21] Chen J., McKay R. M. and Parada L. F. (2012b). Malignant glioma: lessons from genomics, mouse models, and stem cells. *Cell* 149, 36-47. 10.1016/j.cell.2012.03.009 22464322 PMC3719882

[DMM040386C22] Choi P. S. and Meyerson M. (2014). Targeted genomic rearrangements using CRISPR/Cas technology. *Nat. Commun.* 5, 3728 10.1038/ncomms4728 24759083 PMC4170920

[DMM040386C23] Chow L. M. L., Endersby R., Zhu X., Rankin S., Qu C., Zhang J., Broniscer A., Ellison D. W. and Baker S. J. (2011). Cooperativity within and among Pten, p53, and Rb pathways induces high-grade astrocytoma in adult brain. *Cancer Cell* 19, 305-316. 10.1016/j.ccr.2011.01.039 21397855 PMC3060664

[DMM040386C24] Chow R. D., Guzman C. D., Wang G., Schmidt F., Youngblood M. W., Ye L., Errami Y., Dong M. B., Martinez M. A., Zhang S. et al. (2017). AAV-mediated direct in vivo CRISPR screen identifies functional suppressors in glioblastoma. *Nat. Neurosci.* 20, 1329-1341. 10.1038/nn.4620 28805815 PMC5614841

[DMM040386C25] Chung H. K., Jacobs C. L., Huo Y., Yang J., Krumm S. A., Plemper R. K., Tsien R. Y. and Lin M. Z. (2015). Tunable and reversible drug control of protein production via a self-excising degron. *Nat. Chem. Biol.* 11, 713-720. 10.1038/nchembio.1869 26214256 PMC4543534

[DMM040386C26] Conti L., Pollard S. M., Gorba T., Reitano E., Toselli M., Biella G., Sun Y., Sanzone S., Ying Q.-L., Cattaneo E. et al. (2005). Niche-independent symmetrical self-renewal of a mammalian tissue stem cell. *PLoS Biol.* 3, e283 10.1371/journal.pbio.0030283 16086633 PMC1184591

[DMM040386C27] Dahlstrand J., Collins V. P. and Lendahl U. (1992). Expression of the class VI intermediate filament nestin in human central nervous system tumors. *Cancer Res.* 52, 5334-5341. 1382841

[DMM040386C28] deCarvalho A. C., Kim H., Poisson L. M., Winn M. E., Mueller C., Cherba D., Koeman J., Seth S., Protopopov A., Felicella M. et al. (2018). Discordant inheritance of chromosomal and extrachromosomal DNA elements contributes to dynamic disease evolution in glioblastoma. *Nat. Genet.* 50, 708-717. 10.1038/s41588-018-0105-0 29686388 PMC5934307

[DMM040386C29] Dewari P. S., Southgate B., Mccarten K., Monogarov G., O'Duibhir E., Quinn N., Tyrer A., Leitner M.-C., Plumb C., Kalantzaki M. et al. (2018). An efficient and scalable pipeline for epitope tagging in mammalian stem cells using Cas9 ribonucleoprotein. *eLife* 7, 87 10.7554/eLife.35069 PMC5947990 29638216

[DMM040386C30] Ding S., Wu X., Li G., Han M., Zhuang Y. and Xu T. (2005). Efficient transposition of the piggyBac (PB) transposon in mammalian cells and mice. *Cell* 122, 473-483. 10.1016/j.cell.2005.07.013 16096065

[DMM040386C31] Doetsch F., Caillé I., Lim D. A., García-Verdugo J. M. and Alvarez-Buylla A. (1999). Subventricular zone astrocytes are neural stem cells in the adult mammalian brain. *Cell* 97, 703-716. 10.1016/S0092-8674(00)80783-7 10380923

[DMM040386C32] Eckel-Passow J. E., Lachance D. H., Molinaro A. M., Walsh K. M., Decker P. A., Sicotte H., Pekmezci M., Rice T., Kosel M. L., Smirnov I. V. et al. (2015). Glioma groups based on 1p/19q, IDH, and TERT promoter mutations in tumors. *N. Engl. J. Med.* 372, 2499-2508. 10.1056/NEJMoa1407279 26061753 PMC4489704

[DMM040386C33] Funato K., Funato K., Major T., Major T., Lewis P. W., Lewis P. W., Allis C. D., Allis C. D., Tabar V. and Tabar V. (2014). Use of human embryonic stem cells to model pediatric gliomas with H3.3K27M histone mutation. *Science* 346, 1529-1533. 10.1126/science.1253799 25525250 PMC4995593

[DMM040386C34] Furnari F. B., Cloughesy T. F., Cavenee W. K. and Mischel P. S. (2015). Heterogeneity of epidermal growth factor receptor signalling networks in glioblastoma. *Nat. Rev. Cancer* 15, 302-310. 10.1038/nrc3918 25855404 PMC4875778

[DMM040386C35] Galli R., Binda E., Orfanelli U., Cipelletti B., Gritti A., De Vitis S., Fiocco R., Foroni C., DiMeco F. and Vescovi A. (2004). Isolation and characterization of tumorigenic, stem-like neural precursors from human glioblastoma. *Cancer Res.* 64, 7011-7021. 10.1158/0008-5472.CAN-04-1364 15466194

[DMM040386C36] Gallo M., Ho J., Coutinho F. J., Vanner R., Lee L., Head R., Ling E. K. M., Clarke I. D. and Dirks P. B. (2013). A tumorigenic MLL-homeobox network in human glioblastoma stem cells. *Cancer Res.* 73, 417-427. 10.1158/0008-5472.CAN-12-1881 23108137

[DMM040386C37] Gilbertson R. J. and Rich J. N. (2007). Making a tumour's bed: glioblastoma stem cells and the vascular niche. *Nat. Rev. Cancer* 7, 733-736. 10.1038/nrc2246 17882276

[DMM040386C38] Hambardzumyan D. and Bergers G. (2015). Glioblastoma: defining tumor niches. *Trends Cancer* 1, 252-265. 10.1016/j.trecan.2015.10.009 27088132 PMC4831073

[DMM040386C39] Hamilton L., Astell K. R., Velikova G. and Sieger D. (2016). A zebrafish live imaging model reveals differential responses of microglia toward glioblastoma cells in vivo. *Zebrafish* 13, 523-534. 10.1089/zeb.2016.1339 27779463 PMC5124743

[DMM040386C40] Hemmati H. D., Nakano I., Lazareff J. A., Masterman-Smith M., Geschwind D. H., Bronner-Fraser M. and Kornblum H. I. (2003). Cancerous stem cells can arise from pediatric brain tumors. *Proc. Natl. Acad. Sci. USA* 100, 15178-15183. 10.1073/pnas.2036535100 14645703 PMC299944

[DMM040386C41] Holland E. C., Hively W. P., DePinho R. A. and Varmus H. E. (1998). A constitutively active epidermal growth factor receptor cooperates with disruption of G1 cell-cycle arrest pathways to induce glioma-like lesions in mice. *Genes Dev.* 12, 3675-3685. 10.1101/gad.12.23.3675 9851974 PMC317252

[DMM040386C42] Holland E. C., Celestino J., Dai C., Schaefer L., Sawaya R. E. and Fuller G. N. (2000). Combined activation of Ras and Akt in neural progenitors induces glioblastoma formation in mice. *Nat. Genet.* 25, 55-57. 10.1038/75596 10802656

[DMM040386C43] Hsu P. D., Lander E. S. and Zhang F. (2014). Development and applications of CRISPR-Cas9 for genome engineering. *Cell* 157, 1262-1278. 10.1016/j.cell.2014.05.010 24906146 PMC4343198

[DMM040386C44] Hubert C. G., Rivera M., Spangler L. C., Wu Q., Mack S. C., Prager B. C., Couce M., McLendon R. E., Sloan A. E. and Rich J. N. (2016). A three-dimensional organoid culture system derived from human glioblastomas recapitulates the hypoxic gradients and cancer stem cell heterogeneity of tumors found in vivo. *Cancer Res.* 76, 2465-2477. 10.1158/0008-5472.CAN-15-2402 26896279 PMC4873351

[DMM040386C45] Huch M., Knoblich J. A., Lutolf M. P. and Martinez-Arias A. (2017). The hope and the hype of organoid research. *Development* 144, 938-941. 10.1242/dev.150201 28292837

[DMM040386C46] Humpel C. (2015). Organotypic brain slice cultures: a review. *Neuroscience* 305, 86-98. 10.1016/j.neuroscience.2015.07.086 26254240 PMC4699268

[DMM040386C47] Ito H., Nakashima H. and Chiocca E. A. (2019). Molecular responses to immune checkpoint blockade in glioblastoma. *Nat. Med.* 25, 359-361. 10.1038/s41591-019-0385-7 30842671 PMC6742426

[DMM040386C48] Jacob J., Maurange C. and Gould A. P. (2008). Temporal control of neuronal diversity: common regulatory principles in insects and vertebrates? *Development* 135, 3481-3489. 10.1242/dev.016931 18849528

[DMM040386C49] Jacques T. S., Swales A., Brzozowski M. J., Henriquez N. V., Linehan J. M., Mirzadeh Z., O'Malley C., Naumann H., Alvarez-Buylla A. and Brandner S. (2010). Combinations of genetic mutations in the adult neural stem cell compartment determine brain tumour phenotypes. *EMBO J.* 29, 222-235. 10.1038/emboj.2009.327 19927122 PMC2808375

[DMM040386C50] Jiang Y., Marinescu V. D., Xie Y., Jarvius M., Maturi N. P., Haglund C., Olofsson S., Lindberg N., Olofsson T., Leijonmarck C. et al. (2017). Glioblastoma cell malignancy and drug sensitivity are affected by the cell of origin. *Cell Rep.* 18, 977-990. 10.1016/j.celrep.2017.01.003 28122246

[DMM040386C51] Kadoshima T., Sakaguchi H., Nakano T., Soen M., Ando S., Eiraku M. and Sasai Y. (2013). Self-organization of axial polarity, inside-out layer pattern, and species-specific progenitor dynamics in human ES cell-derived neocortex. *Proc. Natl. Acad. Sci. USA* 110, 20284-20289. 10.1073/pnas.1315710110 24277810 PMC3864329

[DMM040386C52] Kaelin W. G. (2017). Common pitfalls in preclinical cancer target validation. *Nat. Rev. Cancer* 17, 425-440. 10.1038/nrc.2017.32 28524181

[DMM040386C53] Khoshnevis M., Carozzo C., Bonnefont-Rebeix C., Belluco S., Leveneur O., Chuzel T., Pillet-Michelland E., Dreyfus M., Roger T., Berger F. et al. (2017). Development of induced glioblastoma by implantation of a human xenograft in Yucatan minipig as a large animal model. *J. Neurosci. Methods* 282, 61-68. 10.1016/j.jneumeth.2017.03.007 28284687

[DMM040386C118] Koehler J. W., Miller A. D., Miller C. R., Porter B., Aldape K., Beck J., Brat D., Cornax I., Corps K., Frank C. et al. (2018). A revised diagnostic classification of canine glioma: towards validation of the canine glioma patient as a naturally occurring preclinical model for human glioma. *J. Neuropathol. Exp. Neurol.* 77, 1039-1054. 10.1093/jnen/nly085 30239918 PMC6181180

[DMM040386C54] Lan X., Jörg D. J., Cavalli F. M. G., Richards L. M., Nguyen L. V., Vanner R. J., Guilhamon P., Lee L., Kushida M. M., Pellacani D. et al. (2017). Fate mapping of human glioblastoma reveals an invariant stem cell hierarchy. *Nature* 549, 227-232. 10.1038/nature23666 28854171 PMC5608080

[DMM040386C55] Lancaster M. A., Renner M., Martin C.-A., Wenzel D., Bicknell L. S., Hurles M. E., Homfray T., Penninger J. M., Jackson A. P. and Knoblich J. A. (2013). Cerebral organoids model human brain development and microcephaly. *Nature* 501, 373-379. 10.1038/nature12517 23995685 PMC3817409

[DMM040386C56] Lee J., Kotliarova S., Kotliarov Y., Li A., Su Q., Donin N. M., Pastorino S., Purow B. W., Christopher N., Zhang W. et al. (2006). Tumor stem cells derived from glioblastomas cultured in bFGF and EGF more closely mirror the phenotype and genotype of primary tumors than do serum-cultured cell lines. *Cancer Cell* 9, 391-403. 10.1016/j.ccr.2006.03.030 16697959

[DMM040386C57] Lee J. H., Lee J. E., Kahng J. Y., Kim S. H., Park J. S., Yoon S. J., Um J.-Y., Kim W. K., Lee J.-K., Park J. et al. (2018). Human glioblastoma arises from subventricular zone cells with low-level driver mutations. *Nature* 560, 243-247. 10.1038/s41586-018-0389-3 30069053

[DMM040386C58] Lendahl U., Zimmerman L. B. and McKay R. D. G. (1990). CNS stem cells express a new class of intermediate filament protein. *Cell* 60, 585-595. 10.1016/0092-8674(90)90662-X 1689217

[DMM040386C59] Liu F., Hon G. C., Villa G. R., Turner K. M., Ikegami S., Yang H., Ye Z., Li B., Kuan S., Lee A. Y. et al. (2015). EGFR mutation promotes glioblastoma through epigenome and transcription factor network remodeling. *Mol. Cell* 60, 307-318. 10.1016/j.molcel.2015.09.002 26455392 PMC4609298

[DMM040386C60] Llorens-Bobadilla E., Zhao S., Baser A., Saiz-Castro G., Zwadlo K. and Martin-Villalba A. (2015). Single-cell transcriptomics reveals a population of dormant neural stem cells that become activated upon brain injury. *Cell Stem Cell* 17, 329-340. 10.1016/j.stem.2015.07.002 26235341

[DMM040386C61] Louis D. N., Perry A., Reifenberger G., von Deimling A., Figarella-Branger D., Cavenee W. K., Ohgaki H., Wiestler O. D., Kleihues P. and Ellison D. W. (2016). The 2016 World Health Organization classification of tumors of the central nervous system: a summary. *Acta Neuropathol.* 131, 803-820. 10.1007/s00401-016-1545-1 27157931

[DMM040386C62] Lu F., Chen Y., Zhao C., Wang H., He D., Xu L., Wang J., He X., Deng Y., Lu E. E. et al. (2016). Olig2-dependent reciprocal shift in pdgf and egf receptor signaling regulates tumor phenotype and mitotic growth in malignant glioma. *Cancer Cell* 29, 669-683. 10.1016/j.ccell.2016.03.027 27165742 PMC4946168

[DMM040386C63] Mack S. C., Hubert C. G., Miller T. E., Taylor M. D. and Rich J. N. (2015). An epigenetic gateway to brain tumor cell identity. *Nat. Neurosci.* 19, 10-19. 10.1038/nn.4190 PMC5568053 26713744

[DMM040386C64] Mackay A., Burford A., Carvalho D., Izquierdo E., Fazal-Salom J., Taylor K. R., Bjerke L., Clarke M., Vinci M., Nandhabalan M. et al. (2017). Integrated molecular meta-analysis of 1,000 pediatric high-grade and diffuse intrinsic pontine glioma. *Cancer Cell* 32, 520-537.e5. 10.1016/j.ccell.2017.08.017 28966033 PMC5637314

[DMM040386C65] Mahne A. E., Mauze S., Joyce-Shaikh B., Xia J., Bowman E. P., Beebe A. M., Cua D. J. and Jain R. (2017). Dual roles for regulatory T-cell depletion and costimulatory signaling in agonistic GITR targeting for tumor immunotherapy. *Cancer Res.* 77, 1108-1118. 10.1158/0008-5472.CAN-16-0797 28122327

[DMM040386C66] Marqués-Torrejón M. Á., Gangoso E. and Pollard S. M. (2017). Modelling glioblastoma tumour-host cell interactions using adult brain organotypic slice co-culture. *Dis. Model. Mech.* 11, dmm031435 10.1242/dmm.031435 PMC5894940 29196443

[DMM040386C67] Marumoto T., Tashiro A., Friedmann-Morvinski D., Scadeng M., Soda Y., Gage F. H. and Verma I. M. (2009). Development of a novel mouse glioma model using lentiviral vectors. *Nat. Med.* 15, 110-116. 10.1038/nm.1863 19122659 PMC2671237

[DMM040386C68] Moreno-Jiménez E. P., Flor-García M., Terreros-Roncal J., Rábano A., Cafini F., Pallas-Bazarra N., Ávila J. and Llorens-Martín M. (2019). Adult hippocampal neurogenesis is abundant in neurologically healthy subjects and drops sharply in patients with Alzheimer's disease. *Nat. Med.* 25, 554-560. 10.1038/s41591-019-0375-9 30911133

[DMM040386C69] Obernier K. and Alvarez-Buylla A. (2019). Neural stem cells: origin, heterogeneity and regulation in the adult mammalian brain. *Development* 146, dev156059 10.1242/dev.156059 30777863 PMC6398449

[DMM040386C70] O'Duibhir E. and Pollard S. M. (2017). Accelerating glioblastoma drug discovery: convergence of patient-derived models, genome editing and phenotypic screening. *Mol. Cell. Neurosci.* 80, 198-207. 10.1016/j.mcn.2016.11.001 27825983 PMC6128397

[DMM040386C71] Ogawa J., Pao G. M., Shokhirev M. N. and Verma I. M. (2018). Glioblastoma model using human cerebral organoids. *Cell Rep.* 23, 1220-1229. 10.1016/j.celrep.2018.03.105 29694897 PMC6892608

[DMM040386C72] Oldrini B., Curiel-García Á., Marques C., Matia V., Uluçkan Ö., Graña-Castro O., Torres-Ruiz R., Rodriguez-Perales S., Huse J. T. and Squatrito M. (2018). Somatic genome editing with the RCAS-TVA-CRISPR-Cas9 system for precision tumor modeling. *Nat. Commun.* 9, 1466 10.1038/s41467-018-03731-w 29654229 PMC5899147

[DMM040386C73] Ottone C., Krusche B., Whitby A., Clements M., Quadrato G., Pitulescu M. E., Adams R. H. and Parrinello S. (2014). Direct cell–cell contact with the vascular niche maintains quiescent neural stem cells. *Nat. Cell Biol.* 16, 1045-1056. 10.1038/ncb3045 25283993 PMC4298702

[DMM040386C74] Parsons D. W., Jones S., Zhang X., Lin J. C.-H., Leary R. J., Angenendt P., Mankoo P., Carter H., Siu I.-M., Gallia G. L. et al. (2008). An integrated genomic analysis of human glioblastoma multiforme. *Science* 321, 1807-1812. 10.1126/science.1164382 18772396 PMC2820389

[DMM040386C75] Pastrana E., Silva-Vargas V. and Doetsch F. (2011). Eyes wide open: a critical review of sphere-formation as an assay for stem cells. *Cell Stem Cell* 8, 486-498. 10.1016/j.stem.2011.04.007 21549325 PMC3633588

[DMM040386C76] Patel A. P., Tirosh I., Trombetta J. J., Shalek A. K., Gillespie S. M., Wakimoto H., Cahill D. P., Nahed B. V., Curry W. T., Martuza R. L. et al. (2014). Single-cell RNA-seq highlights intratumoral heterogeneity in primary glioblastoma. *Science* 344, 1396-1401. 10.1126/science.1254257 24925914 PMC4123637

[DMM040386C77] Pathania M., De Jay N., Maestro N., Harutyunyan A. S., Nitarska J., Pahlavan P., Henderson S., Mikael L. G., Richard-Londt A., Zhang Y. et al. (2017). H3.3K27M cooperates with Trp53 loss and PDGFRA gain in mouse embryonic neural progenitor cells to induce invasive high-grade gliomas. *Cancer Cell* 32, 684-700.e9. 10.1016/j.ccell.2017.09.014 29107533 PMC5687892

[DMM040386C78] Piccirillo S. G. M., Colman S., Potter N. E., van Delft F. W., Lillis S., Carnicer M.-J., Kearney L., Watts C. and Greaves M. (2015). Genetic and functional diversity of propagating cells in glioblastoma. *Stem Cell Rep.* 4, 7-15. 10.1016/j.stemcr.2014.11.003 PMC4297869 25533637

[DMM040386C79] Plog B. A. and Nedergaard M. (2018). The glymphatic system in central nervous system health and disease: past, present, and future. *Annu. Rev. Pathol.* 13, 379-394. 10.1146/annurev-pathol-051217-111018 29195051 PMC5803388

[DMM040386C80] Pollard S. M., Yoshikawa K., Clarke I. D., Danovi D., Stricker S., Russell R., Bayani J., Head R., Lee M., Bernstein M. et al. (2009). Glioma stem cell lines expanded in adherent culture have tumor-specific phenotypes and are suitable for chemical and genetic screens. *Cell Stem Cell* 4, 568-580. 10.1016/j.stem.2009.03.014 19497285

[DMM040386C81] Pollen A. A., Nowakowski T. J., Chen J., Retallack H., Sandoval-Espinosa C., Nicholas C. R., Shuga J., Liu S. J., Oldham M. C., Diaz A. et al. (2015). Molecular identity of human outer radial glia during cortical development. *Cell* 163, 55-67. 10.1016/j.cell.2015.09.004 26406371 PMC4583716

[DMM040386C82] Pontén J. and Macintyre E. H. (1968). Long term culture of normal and neoplastic human glia. *Acta Pathol. Microbiol. Scand.* 74, 465-486. 10.1111/j.1699-0463.1968.tb03502.x 4313504

[DMM040386C89] Prykhozhij S. V. and Berman J. N. (2018). Zebrafish knock-ins swim into the mainstream. *Dis. Model. Mech.* 11, dmm037515 10.1242/dmm.037515 30366936 PMC6215421

[DMM040386C83] Pudelko L., Edwards S., Balan M., Nyqvist D., Al-Saadi J., Dittmer J., Almlöf I., Helleday T. and Bräutigam L. (2018). An orthotopic glioblastoma animal model suitable for high-throughput screenings. *Neuro Oncol.* 20, 1475-1484. 10.1093/neuonc/noy071 29750281 PMC6176805

[DMM040386C84] Quail D. F. and Joyce J. A. (2017). The microenvironmental landscape of brain tumors. *Cancer Cell* 31, 326-341. 10.1016/j.ccell.2017.02.009 28292436 PMC5424263

[DMM040386C85] Ray J., Peterson D. A., Schinstine M. and Gage F. H. (1993). Proliferation, differentiation, and long-term culture of primary hippocampal neurons. *Proc. Natl. Acad. Sci. USA* 90, 3602-3606. 10.1073/pnas.90.8.3602 8475109 PMC46349

[DMM040386C86] Read R. D., Cavenee W. K., Furnari F. B. and Thomas J. B. (2009). A drosophila model for EGFR-Ras and PI3K-dependent human glioma. *PLoS Genet.* 5, e1000374 10.1371/journal.pgen.1000374 19214224 PMC2636203

[DMM040386C87] Reynolds B. A. and Weiss S. (1992). Generation of neurons and astrocytes from isolated cells of the adult mammalian central nervous system. *Science* 255, 1707-1710. 10.1126/science.1553558 1553558

[DMM040386C88] Sanai N., Tramontin A. D., Quiñones-Hinojosa A., Barbaro N. M., Gupta N., Kunwar S., Lawton M. T., McDermott M. W., Parsa A. T., Verdugo J. M.-G. et al. (2004). Unique astrocyte ribbon in adult human brain contains neural stem cells but lacks chain migration. *Nature* 427, 740-744. 10.1038/nature02301 14973487

[DMM040386C90] Schiffer D., Giordana M. T., Pezzotta S., Lechner C. and Paoletti P. (1978). Cerebral tumors induced by transplacental ENU: study of the different tumoral stages, particularly of early proliferations. *Acta Neuropathol.* 41, 27-31. 10.1007/BF00689553 636834

[DMM040386C91] Schwartzentruber J., Korshunov A., Liu X.-Y., Jones D. T. W., Pfaff E., Jacob K., Sturm D., Fontebasso A. M., Quang D.-A. K., Tönjes M. et al. (2012). Driver mutations in histone H3.3 and chromatin remodelling genes in paediatric glioblastoma. *Nature* 482, 226-231. 10.1038/nature10833 22286061

[DMM040386C92] Shultz L. D., Brehm M. A., Garcia-Martinez J. V. and Greiner D. L. (2012). Humanized mice for immune system investigation: progress, promise and challenges. *Nat. Rev. Immunol.* 12, 786-798. 10.1038/nri3311 23059428 PMC3749872

[DMM040386C93] Singh S. K., Clarke I. D., Terasaki M., Bonn V. E., Hawkins C., Squire J. and Dirks P. B. (2003). Identification of a cancer stem cell in human brain tumors. *Cancer Res.* 63, 5821-5828. 14522905

[DMM040386C94] Singh S. K., Singh S. K., Hawkins C., Hawkins C., Clarke I. D., Clarke I. D., Squire J. A., Squire J. A., Bayani J., Bayani J. et al. (2004). Identification of human brain tumour initiating cells. *Nature* 432, 396-401. 10.1038/nature03128 15549107

[DMM040386C95] Singh D. K., Kollipara R. K., Vemireddy V., Yang X.-L., Sun Y., Regmi N., Klingler S., Hatanpaa K. J., Raisanen J., Cho S. K. et al. (2017). Oncogenes activate an autonomous transcriptional regulatory circuit that drives glioblastoma. *Cell Rep.* 18, 961-976. 10.1016/j.celrep.2016.12.064 28122245 PMC5321610

[DMM040386C96] Snuderl M., Fazlollahi L., Le L. P., Nitta M., Zhelyazkova B. H., Davidson C. J., Akhavanfard S., Cahill D. P., Aldape K. D., Betensky R. A. et al. (2011). Mosaic amplification of multiple receptor tyrosine kinase genes in glioblastoma. *Cancer Cell* 20, 810-817. 10.1016/j.ccr.2011.11.005 22137795

[DMM040386C97] Sorrells S. F., Paredes M. F., Cebrian-Silla A., Sandoval K., Qi D., Kelley K. W., James D., Mayer S., Chang J., Auguste K. I. et al. (2018). Human hippocampal neurogenesis drops sharply in children to undetectable levels in adults. *Nature* 555, 377-381. 10.1038/nature25975 29513649 PMC6179355

[DMM040386C98] Sousa-Nunes R., Cheng L. Y. and Gould A. P. (2010). Regulating neural proliferation in the Drosophila CNS. *Curr. Opin. Neurobiol.* 20, 50-57. 10.1016/j.conb.2009.12.005 20079625

[DMM040386C99] Stewart A. M., Braubach O., Spitsbergen J., Gerlai R. and Kalueff A. V. (2014). Zebrafish models for translational neuroscience research: from tank to bedside. *Trends Neurosci.* 37, 264-278. 10.1016/j.tins.2014.02.011 24726051 PMC4039217

[DMM040386C100] Stupp R., Mason W. P., van den Bent M. J., Weller M., Fisher B., Taphoorn M. J. B., Belanger K., Brandes A. A., Marosi C., Bogdahn U. et al. (2005). Radiotherapy plus concomitant and adjuvant temozolomide for glioblastoma. *N. Engl. J. Med.* 352, 987-996. 10.1056/NEJMoa043330 15758009

[DMM040386C101] Sturm D., Witt H., Hovestadt V., Khuong-Quang D.-A., Jones D. T. W., Konermann C., Pfaff E., Tönjes M., Sill M., Bender S. et al. (2012). Hotspot mutations in H3F3A and IDH1 define distinct epigenetic and biological subgroups of glioblastoma. *Cancer Cell* 22, 425-437. 10.1016/j.ccr.2012.08.024 23079654

[DMM040386C102] Sun Y., Pollard S., Conti L., Toselli M., Biella G., Parkin G., Willatt L., Falk A., Cattaneo E. and Smith A. (2008). Long-term tripotent differentiation capacity of human neural stem (NS) cells in adherent culture. *Mol. Cell. Neurosci.* 38, 245-258. 10.1016/j.mcn.2008.02.014 18450476

[DMM040386C103] Suva M. L., Riggi N. and Bernstein B. E. (2013). Epigenetic reprogramming in cancer. *Science* 339, 1567-1570. 10.1126/science.1230184 23539597 PMC3821556

[DMM040386C104] Suvà M. L., Rheinbay E., Gillespie S. M., Patel A. P., Wakimoto H., Rabkin S. D., Riggi N., Chi A. S., Cahill D. P., Nahed B. V. et al. (2014). Reconstructing and reprogramming the tumor-propagating potential of glioblastoma stem-like cells. *Cell* 157, 580-594. 10.1016/j.cell.2014.02.030 24726434 PMC4004670

[DMM040386C105] Szatmári T., Lumniczky K., Désaknai S., Trajcevski S., Hídvégi E. J., Hamada H. and Sáfrány G. (2006). Detailed characterization of the mouse glioma 261 tumor model for experimental glioblastoma therapy. *Cancer Sci.* 97, 546-553. 10.1111/j.1349-7006.2006.00208.x 16734735 PMC11159227

[DMM040386C106] Temple S. (1989). Division and differentiation of isolated CNS blast cells in microculture. *Nature* 340, 471-473. 10.1038/340471a0 2755510

[DMM040386C107] Toledo C. M., Ding Y., Hoellerbauer P., Davis R. J., Basom R., Girard E. J., Lee E., Corrin P., Hart T., Bolouri H. et al. (2015). Genome-wide CRISPR-Cas9 screens reveal loss of redundancy between PKMYT1 and WEE1 in glioblastoma stem-like cells. *Cell Rep.* 13, 2425-2439. 10.1016/j.celrep.2015.11.021 26673326 PMC4691575

[DMM040386C108] Turner K. M., Deshpande V., Beyter D., Koga T., Rusert J., Lee C., Li B., Arden K., Ren B., Nathanson D. A. et al. (2017). Extrachromosomal oncogene amplification drives tumour evolution and genetic heterogeneity. *Nature* 543, 122-125. 10.1038/nature21356 28178237 PMC5334176

[DMM040386C109] Tuveson D. and Clevers H. (2019). Cancer modeling meets human organoid technology. *Science* 364, 952-955. 10.1126/science.aaw6985 31171691

[DMM040386C110] Uchida N., Buck D. W., He D., Reitsma M. J., Masek M., Phan T. V., Tsukamoto A. S., Gage F. H. and Weissman I. L. (2000). Direct isolation of human central nervous system stem cells. *Proc. Natl. Acad. Sci. USA* 97, 14720-14725. 10.1073/pnas.97.26.14720 11121071 PMC18985

[DMM040386C111] Verhaak R. G. W., Hoadley K. A., Purdom E., Wang V., Qi Y., Wilkerson M. D., Miller C. R., Ding L., Golub T., Mesirov J. P. et al. (2010). Integrated genomic analysis identifies clinically relevant subtypes of glioblastoma characterized by abnormalities in PDGFRA, IDH1, EGFR, and NF1. *Cancer Cell* 17, 98-110. 10.1016/j.ccr.2009.12.020 20129251 PMC2818769

[DMM040386C112] Verhaak R. G. W., Bafna V. and Mischel P. S. (2019). Extrachromosomal oncogene amplification in tumour pathogenesis and evolution. *Nat. Rev. Cancer* 19, 283-288. 10.1038/s41568-019-0128-6 30872802 PMC7168519

[DMM040386C113] Wang Q., Hu B., Hu X., Kim H., Squatrito M., Scarpace L., deCarvalho A. C., Lyu S., Li P., Li Y. et al. (2017). Tumor evolution of glioma-intrinsic gene expression subtypes associates with immunological changes in the microenvironment. *Cancer Cell* 32, 42-56.e6. 10.1016/j.ccell.2017.06.003 28697342 PMC5599156

[DMM040386C114] Wright A. V., Nuñez J. K. and Doudna J. A. (2016). Biology and applications of CRISPR systems: harnessing nature's toolbox for genome engineering. *Cell* 164, 29-44. 10.1016/j.cell.2015.12.035 26771484

[DMM040386C115] Xie Y., Bergström T., Jiang Y., Johansson P., Marinescu V. D., Lindberg N., Segerman A., Wicher G., Niklasson M., Baskaran S. et al. (2015). The human glioblastoma cell culture resource: validated cell models representing all molecular subtypes. *EBioMedicine* 2, 1351-1363. 10.1016/j.ebiom.2015.08.026 26629530 PMC4634360

[DMM040386C116] Zhu Y., Zhu Y., Guignard F., Guignard F., Zhao D., Zhao D., Liu L., Liu L., Burns D. K., Burns D. K. et al. (2005). Early inactivation of p53 tumor suppressor gene cooperating with NF1 loss induces malignant astrocytoma. *Cancer Cell* 8, 119-130. 10.1016/j.ccr.2005.07.004 16098465 PMC3024718

